# The value of nucleoporin 188 in diagnosis, prognosis and immunoregulation: from pan-cancer analysis to gastric cancer verification

**DOI:** 10.3389/fimmu.2025.1586784

**Published:** 2025-08-08

**Authors:** Zhengfeng Zhu, Ru Peng, Huanhuan Cai

**Affiliations:** ^1^ Department of Medical Oncology, JianHu People’s Hospital, Jianhu, China; ^2^ School of Clinical Medicine, Jiangsu Health Vocational College, Nanjing, China; ^3^ Department of Basic Medicine, Suzhou Vocational Health College, Suzhou, China

**Keywords:** nucleoporin 188, diagnosis, prognosis, immunoregulation, pan-cancer, gastric cancer

## Abstract

**Background:**

Nucleoporin 188 (NUP188) is a vital component of the nuclear pore complex that regulates cancer progression, but its role in diagnosis, prognosis, and immunoregulation in pan-cancer remains unclear.

**Methods:**

A comprehensive exploration of the value of NUP188 was performed using different platforms and software, including TCGA, HPA, UALCAN, TISIDB, cBioPortal, STRING, GSCALite, cancerSEA databases, and R software. The expression of NUP188 in gastric cancer (GC) was detected by immunohistochemistry. The association between NUP188 and clinicopathologic features was explored, and the predictive value of NUP188 was determined. The effect of NUP188 knockdown on the proliferation, migration, and invasion of GC cells was detected *in vitro* and vivo.

**Results:**

NUP188 was dysregulated in most human cancers compared to normal tissues with great diagnostic accuracy. The NUP188 expression was associated with molecular subtype, pathologic stage, prognosis, cancer status, and genomic heterogeneity in multiple cancer types. NUP188 was also related to the immune subtype and infiltrating levels of immune cells. The protein level of NUP188 was both upregulated in GC tissues, and was related to HP infection, depth of invasion, distant metastasis, TNM stage, and overall survival of GC patients. NUP188 interference restrained the proliferation, migration, and invasion of GC cells, and inhibited the GC growth *in vivo*.

**Conclusion:**

NUP188 plays a potential role in pan-cancer diagnosis and prognosis and may serve as a novel biomarker for tumor immunotherapy.

## 1 Introduction

Nuclear pore complexes (NPCs) are the only pores embedded in the nuclear membrane that control material transport between the nucleus and the cytoplasm. NPC also regulates biological functions such as RNA processing, DNA repair, transcriptional regulation, and cell cycle regulation (1). NPC is essential in maintaining normal cell signaling and protein localization, closely related to carcinogenesis (2). The NPC is made up of approximately 30 distinct proteins called nucleoporins. Multiple nucleoporins have been confirmed to be involved in the occurrence and development of tumors, such as NUP98, NUP62, and NUP88 (3). Therefore, nucleoporins are gradually becoming a hot spot in current research.

Nucleoporin 188 (NUP188) is a member of the NUP93 complex, showing a significant impact on NPC formation. Theerthagiri et al. confirmed that decreased NUP188 accelerated the translocation of integral membrane proteins by regulating NPCs’ function (4). Knockdown of NUP188 also hinders chromosome segregation, resulting in mitotic arrest (5). In addition, NUP188 mutation might relate to a new developmental syndrome with a poor prognosis (6). However, little is known about NUP188s role in cancer progression.

This study systematically explored the NUP188 expression in pan-cancer, and its prognostic value was analyzed comprehensively. The connections between NUP188, cancer status, genomic heterogeneity, and infiltrating lymphocytes were also explored. Finally, immunohistochemistry detected the NUP188 expression in gastric cancer tissues. The influence of NUP188 on GC cells was detected after NUP188 knockdown *in vitro*. The study aimed to demonstrate the potential of NUP188 in cancer diagnosis, prognosis, and immunoregulation, thus affording a new insight into tumor therapy.

## 2 Materials and methods

### 2.1 The NUP188 expression in pan-cancer

The NUP188 expression in 33 tumor tissues and corresponding non-tumor tissues was downloaded from the TCGA database (https://portal.gdc.cancer.gov) after sequence alignment had been completed using the STAR software. Batch effects were corrected using the “ComBat” function from the “sva” package. TPM normalization was conducted using the “edgeR” package with “calcNormFactors” function (parameter method = “TMM”), and the data was transformed using the log2(value +1) normalization method. According to the characteristics of the data format, the appropriate statistical methods (R package “stats” and “car”) were selected for statistics, and the “ggplot2” was used to visualize the data. The NUP188 protein expression in pan-cancer was explored in the UALCAN database (https://ualcan.path.uab.edu/). The representative pictures of NUP188 in different tissues were afforded by the HPA database (https://www.proteinatlas.org/).

### 2.2 The receiver operating characteristic curve of NUP188 in human cancers

The RNAseq downloaded from the TCGA database was utilized to perform the ROC analysis through the R package “pROC”, and the results were visualized by the “ggplot2”. The pROC package automatically adjusted the order of outcomes for the data by default.

### 2.3 The prognostic analysis of NUP188 in pan-cancer

The NUP188 expression in different molecular subtypes was confirmed by the TISIDB database (http://cis.hku.hk/TISIDB/index.php). The relationship between NUP188 and the pathologic stage was explored by the R package “stats” based on the RNAseq from the TCGA database. Proportional risk hypothesis testing and Cox regression analysis were performed using the package “survival”, and forest map visualization was performed using “ggplot2”.

### 2.4 The gene functions of NUP188 in different cancers

The UNP188-related genes were calculated with high confidence (0.700) by the STRING database (https://string-db.org/), and then enrichment analysis was performed by the package “clusterProfiler”. The cellular processes activated or inhibited by NUP188 in 32 cancers was detected by the GSCALite database (https://guolab.wchscu.cn/GSCA/#/). The relationship between NUP188 and cancer cell status was explored by the cancerSEA database (http://biocc.hrbmu.edu.cn/CancerSEA/).

### 2.5 Genomic heterogeneity analysis of NUP188 in pan-cancer

NUP188 mutations in human cancers were demonstrated by the cBioPortal database (https://www.cbioportal.org/). The mutant landscape of NUP188 in GC was demonstrated by the Sangerbox platform (http://vip.sangerbox.com/). The correlations between NUP188 and tumor mutation burden (TMB), microsatellite instability (MSI), ploidy, mutant-allele tumor heterogeneity (MATH), neoantigen (Neo), homologous recombination deficiency (HRD) and loss of heterozygosity (LOH) were also calculated by the Sangerbox platform with filtering criteria: excluding silent mutations, germline variants, and variants with VAF <5%.

### 2.6 The immune analysis of NUP188 in human cancers

The NUP188 expression in different immune subtypes of different cancers was detected by the TISIDB database. The uniformly standardized pan-cancer dataset TCGA TARGET GTEx was downloaded from the UCSC database (https://xenabrowser.net/). The expression data of the NUP188 gene in each sample were then extracted. Each expression value was further subjected to a log2(value + 0.001) transformation. Additionally, the gene expression profiles of each tumor were extracted from this dataset and mapped to GeneSymbols. Furthermore, the stromal, immune, and ESTIMATE scores for each patient in each tumor were calculated using the R package “ESTIMATE” based on the gene expression. The immune infiltration score was calculated through the R package “GSVA” according to the markers of immune cells provided in the literature (7). The correlations between NUP188 and immune cells were explored through the Pearson methods.

### 2.7 Tissue samples

A total of 410 patients who underwent biopsy or surgery at JianHu People’s Hospital between December 2015 and May 2019 participated in the study, providing 410 GC tissues and 98 para-carcinoma tissues. Patients ranged in age from 32 to 76 years, with an average age of 53. The Ethics Committee of JianHu People’s Hospital approved this study (JHYY20200045), and each patient or the guardian signed the written consents.

### 2.8 Immunohistochemistry

The IHC was conducted according to the instruction of the Broad Spectrum SP Kit (SP0041, Solarbio, China). The NUP188 in tissues was detected by Rabbit polyclonal anti-NUP188 (ab204490, Abcam, USA). Two pathologists evaluated IHC results in a double-blind setting. The staining intensity of NUP188 in cells of GC tissues was rated by a semi-quantitative score method (8). Based on the survival of GC patients, a cut-off value of 190 was calculated using X⁃tile software, and NUP188 expression was divided into high expression and low or no expression.

### 2.9 Experimental methods

The experimental methods are shown in **Supplementary Methods**.

## 3 Results

### 3.1 The expression of NUP188 in human cancers

**Figure 1:**
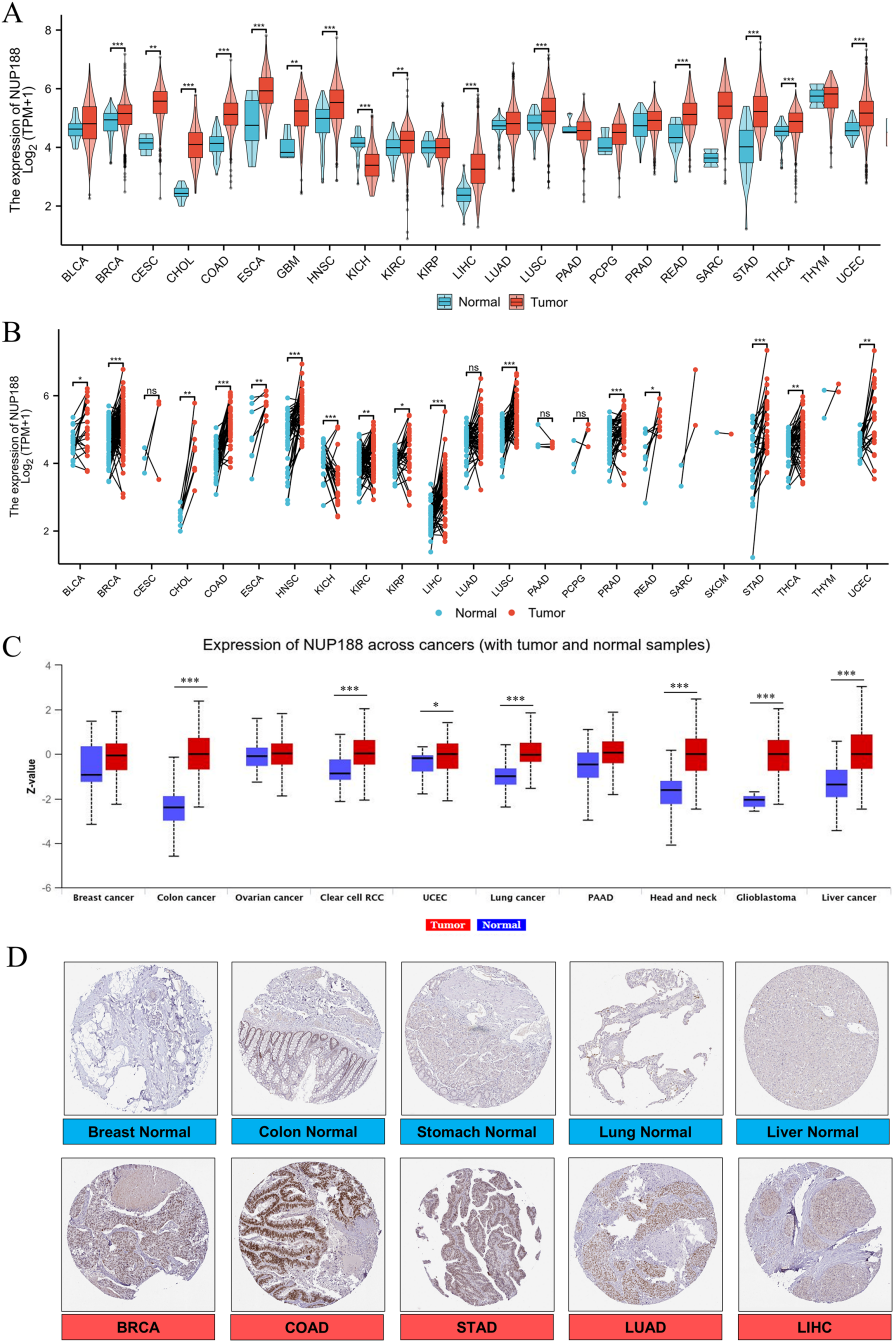
NUP188 expression in cancer tissues. **(A)** NUP188 mRNA expression in different human cancers and normal tissues based on the TCGA database. **(B)** NUP188 mRNA expression in paired human cancers and normal tissues based on the TCGA database. **(C)** NUP188 protein expression in different human cancers and normal tissues based on the UALCAN database. **(D)** The representative pictures of NUP188 protein in cancer tissues and normal tissues based on the HPA database. *P<0.05, **P<0.01, ***P<0.001.

**Figure 2:**
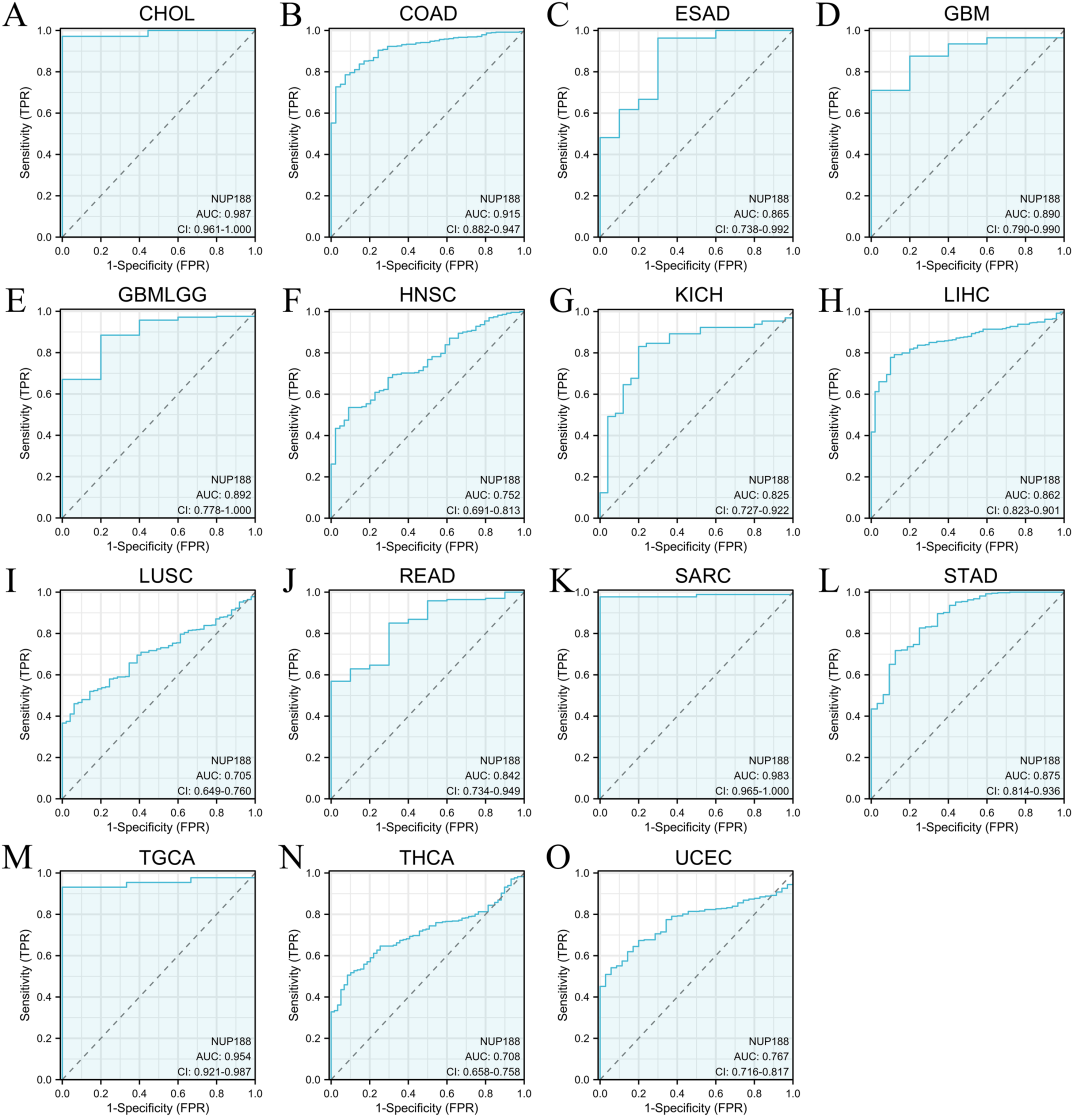
The ROC curves of NUP188 in human cancers. **(A)** CHOL. **(B)** COAD. **(C)** ESAD. **(D)** GBM. **(E)** GBMLGG. **(F)** HNSC. **(G)** KICH. **(H)** LIHC. **(I)** LUSC. **(J)** READ. **(K)** SARC. **(L)** STAD. **(M)** TGCA. **(N)** THCA. **(O)** UCEC.

The RNAseq of the TCGA database demonstrated that NUP188 mRNA was unregulated in 14 cancers and only downregulated in kidney chromophobe (KICH) compared to normal tissues (**Figure 1A**). The paired sample t-test further confirmed that NUP188 mRNA overexpression was found in 15 cancers except KICH (**Figure 1B**). Similarly, the UALCAN database confirmed that NUP188 protein level was overexpressed in colon cancer, clear cell renal cell carcinoma (RCC), uterine corpus endometrial carcinoma (UCEC), lung cancer, head and neck squamous cell carcinoma (HNSC), glioblastoma, and liver cancer (**Figure 1C**). As shown in **Figure 1D**, NUP188 was localized to the nuclear, and a relatively strong positive staining was present in some samples of cancer tissues. Meanwhile, NUP188 showed potential diagnostic accuracy in 15 cancers, with the area under the ROC curve greater than 0.7 (**Figure 2**). These results indicated that NUP188 might be a novel biomarker for cancer diagnosis.

### 3.2 The association between NUP188 and prognosis in different cancers

**Figure 3:**
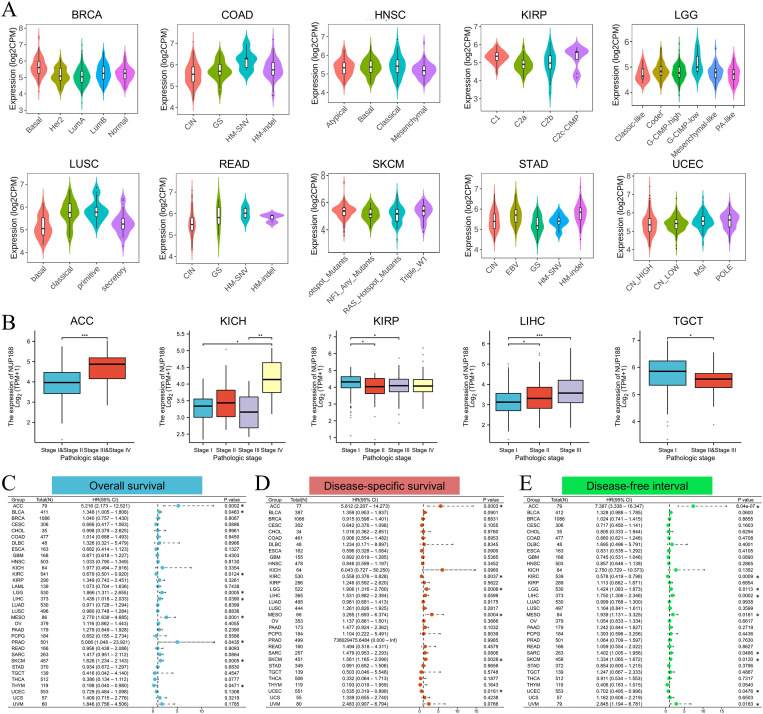
The relationships between NUP188 and clinical significance. **(A)** The levels of NUP188 in different molecular subtypes of human cancers. **(B)** The levels of NUP188 in different pathologic stages of 5 cancers. **(C-E)** The role of NUP188 in overall survival **(C)**, disease-specific survival **(D)** and disease-free interval **(E)**. *P<0.05, **P<0.01, ***P<0.001.

The TISIDB database demonstrated that different molecular subtypes showed different NUP188 expression in 10 cancer types (**Figure 3A**). Furthermore, advanced pathologic stages showed higher NUP188 levels in adrenocortical carcinoma (ACC), KICH, and liver hepatocellular carcinoma (LIHC) while presented lower NUP188 levels in kidney renal papillary cell carcinoma (KIRP) and testicular germ cell tumors (TGCT) (**Figure 3B**). Cox regression analysis was performed to evaluate the prognostic value of NUP188 in human cancers. In overall survival (OS), NUP188 was a risk factor for patients with ACC, bladder urothelial carcinoma (BLCA), brain lower grade glioma (LGG), LIHC, mesothelioma (MESO), and skin cutaneous melanoma (SKCM). It worked as a protective factor for patients with kidney renal clear cell carcinoma (KIRC) and thymoma (THYM) (**Figure 3C**). In disease-specific survival (DSS), high NUP188 expression might predict poor prognosis in ACC, LGG, MESO, and SKCM, but indicate good news in KIRC and UCEC (**Figure 3D**). In the disease-free interval (DFI), NUP188 played a risk role for patients with ACC, LGG, LIHC, MESO, sarcoma (SARC), SKCM, and uveal melanoma (UVM), but benefited the patients with KIRC and UCEC (**Figure 3E**). These results suggested that NUP188 regulated the tumor progression and affected the prognosis in multiple cancer types.

### 3.3 The gene functions of NUP188 in pan-cancer

**Figure 4:**
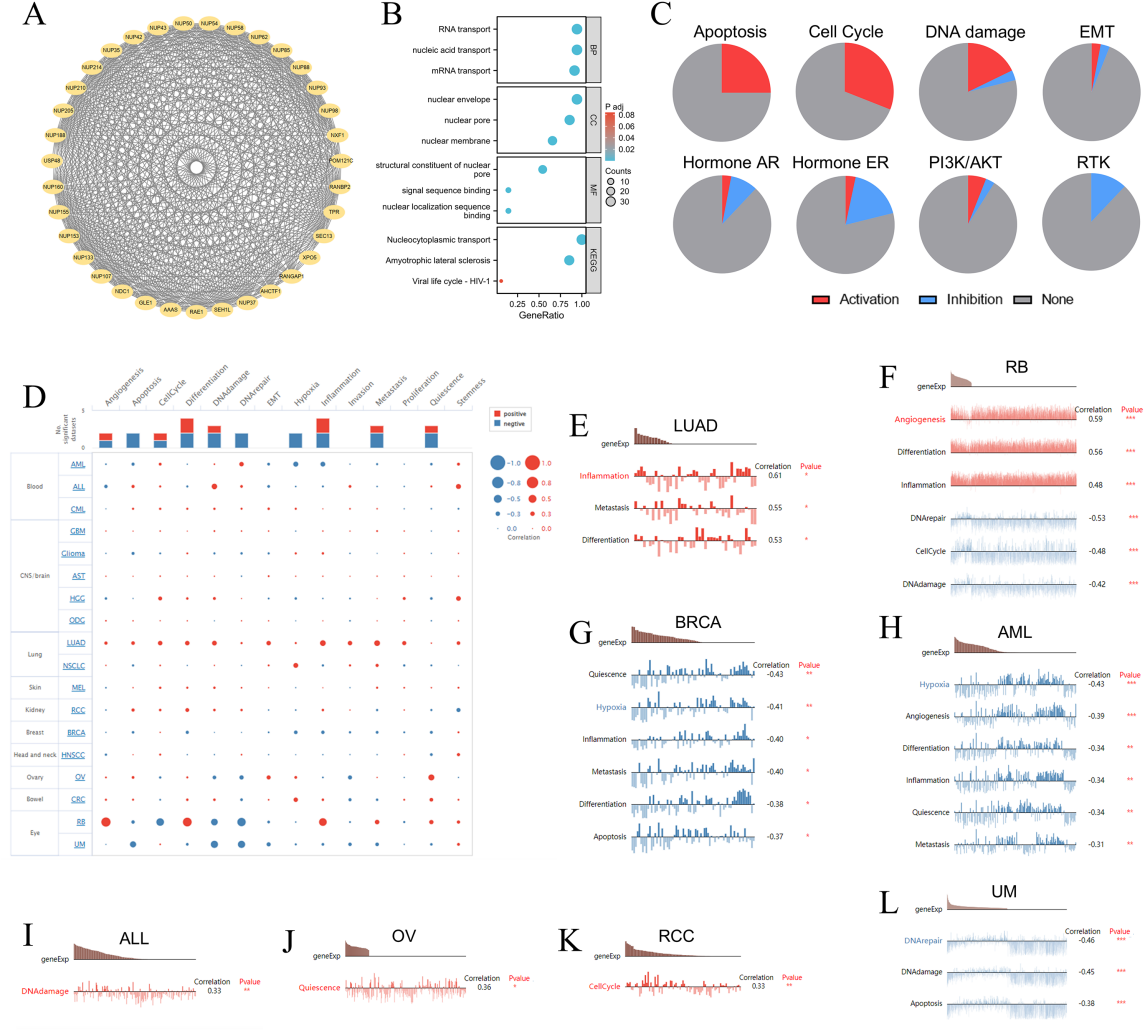
The gene functions of NUP188 in pan-cancer. **(A)** The PPI network around NUP188 based on the STRING database. **(B)** The GO and KEGG enrichment analysis of the top proteins related to NUP188. **(C)** The processes activated or inhibited by NUP188 in the GSCALite database. **(D)** The interactive bubble chart of connections between NUP188 and functional status in the CancerSEA database. **(E-L)** The relationships between NUP188 expression and functional statuses in **(E)** LUAD, **(F)** RB, **(G)** BRCA, **(H)** AML, **(I)** ALL, **(J)** OV, **(K)** RCC, **(L)** UM. *P<0.05, **P<0.01, ***P<0.001.

The STRING database identified the 34 NUP188-related proteins with high confidence (>0.7) (**Figure 4A**), and the GO/KEGG analysis of these proteins demonstrated that the functions of NUP188 might be mainly nucleic acid transport and NPC formation (**Figure 4B**). The GSCALite database showed the biological functions and pathways associated with NUP188 in 32 cancers. The result confirmed that NUP188 activated the apoptosis, cell cycle, and DNA damage in multiple cancer types, but inhibited the signaling pathways like hormone ER and RTK in some cancers (**Figure 4C**). Subsequently, we explore the influence of NUP188 on the functional status of cancer cell at the single-cell level through the cancerSEA database (**Figure 4D**). Specifically, NUP188 was positively associated with inflammation, metastasis, and differentiation in lung adenocarcinoma (LUAD), with angiogenesis, differentiation, and inflammation in RB, with DNA damage in ALL, with quiescence in OV, with cell cycle in RCC, while showed negative connection to DNA repair, cell cycle, and DNA damage in retinoblastoma (RB), to quiescence, hypoxia, inflammation, metastasis, differentiation, and apoptosis in breast invasive carcinoma (BRCA), to hypoxia, angiogenesis, differentiation, inflammation, quiescence, and metastasis in acute myeloid leukemia (AML), to DNA repair, DNA damage, and apoptosis in uveal melanoma (UM) (**Figures 4E–L**). These results demonstrated that NUP188 might regulate cancer progression by affecting the biological behaviors of cancer cells.

### 3.4 The connection between NUP188 and genomic heterogeneity in pan-cancer

**Figure 5:**
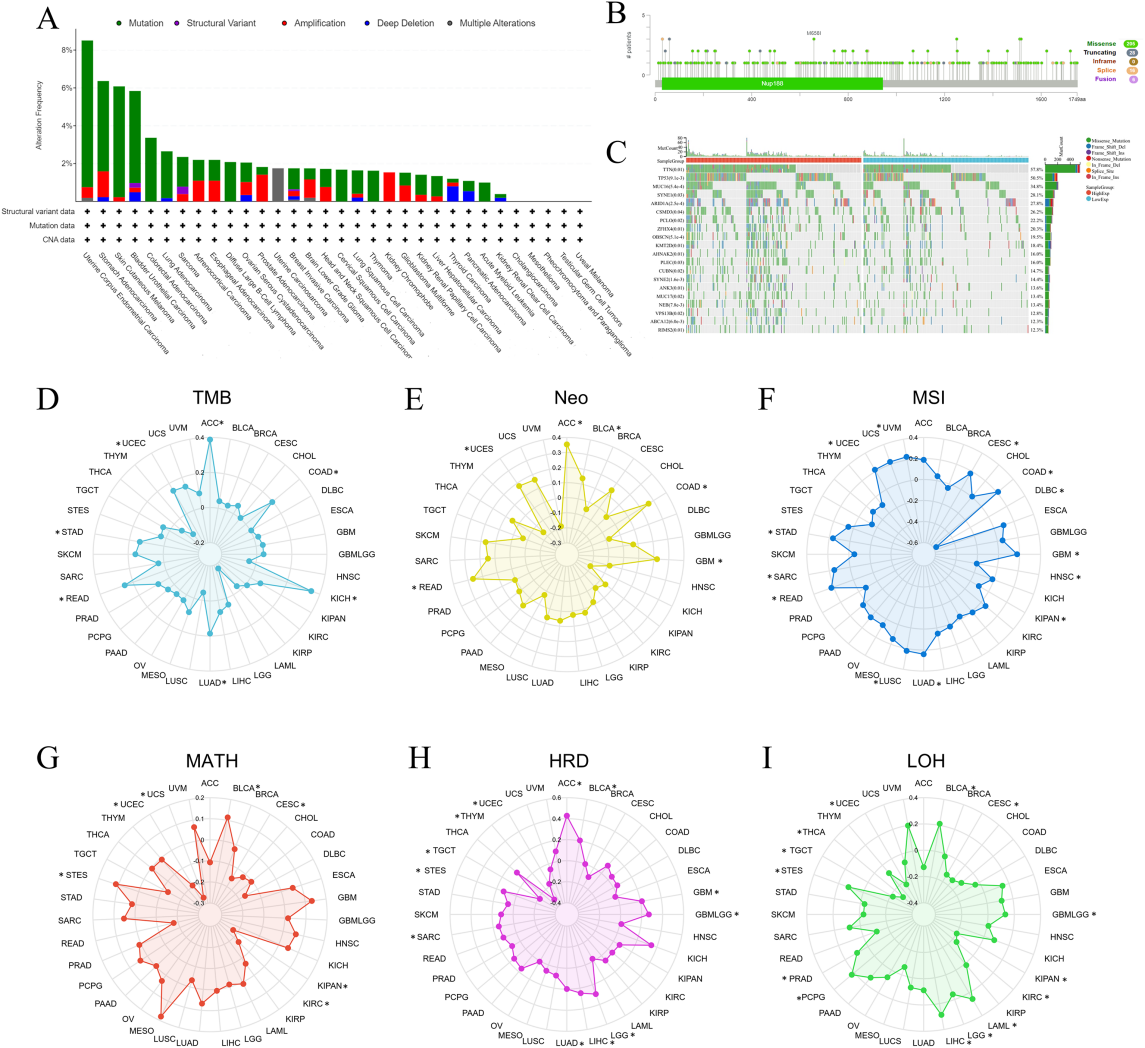
The associations between NUP188 and genomic heterogeneity in pan-cancer. **(A)** Bar chart of NUP188 mutations in human cancers. **(B)** Mutation diagram of NUP188 across protein domains. **(C)** The top 15 genes with the highest frequency of mutations in the high NUP188 group and low NUP188 group in STAD. The relationship between NUP188 and TMB **(D)**, Meo **(E)**, MSI **(F)**, MATH **(G)**, HRD **(H)**, and LOH **(I)**. *P<0.05.

Genomic heterogeneity is an important index in measuring tumor characteristics, and we first explored the NUP188 alterations in pan-cancer via the cBioPortal database. The mutation was the main alteration, especially in UCEC, stomach adenocarcinoma (STAD), SKCM, and bladder urothelial carcinoma (**Figure 5A**). A total of 255 mutation sites were found in 1749 amino acids, including 205 missenses, 28 truncating, and 16 splices, and M658I was the most common mutation site (**Figure 5B**). Furthermore, the mutation landscape of NUP188 identified the mutated genes in STAD, and the top 5 genes were TTN, TP53, MUC16, SYNE1 and ARIDIA (**Figure 5C**).

Genomic heterogeneity was associated with the prognosis and therapy efficacy, and then we evaluated the correlations between NUP188 and 6 indexes. NUP188 showed a positive relationship with TMB in LUAD, colon adenocarcinoma (COAD), STAD, UCEC, rectum adenocarcinoma (READ), ACC, and KICH (**Figure 5D**). There were positive associations between NUP188 and Neo in glioblastoma multiforme (GBM), COAD, READ, UCEC, BLCA, and ACC (**Figure 5E**). For MSI, NUP 188 was a positively related factor in GBM, CESC, LUAD, COAD, SARC, STAD, UCEC, LUSC, READ, and UVM, but played a negatively related role in KIPAN, head and neck squamous cell carcinoma (HNSC), and lymphoid neoplasm diffuse large B-cell lymphoma (DLBC) (**Figure 5F**). NUP188 was positively connected to MATH in STES and BLCA, and negatively in CESC, KIPAN, UCEC, KIRC and UCS (**Figure 5G**). NUP188 presented a positive association with HRD in GBM, GBMLGG, LGG, LUAD, stomach and Esophageal carcinoma (STES), SARC, LIHC, BLCA, and ACC. It showed negative connection in UCEC, THYM and TGCT (**Figure 5H**). Finally, NUP188 was positively related to LOH in glioma (GBMLGG), LGG, LAML, STES, PRAD, LIHC, PCPG, and BLCA, and negatively in CESC, KIPAN, UCEC, KIRC, THCA, and TGCT (**Figure 5I**).

### 3.5 The relationship between NUP188 and infiltrating immune cells

**Figure 6:**
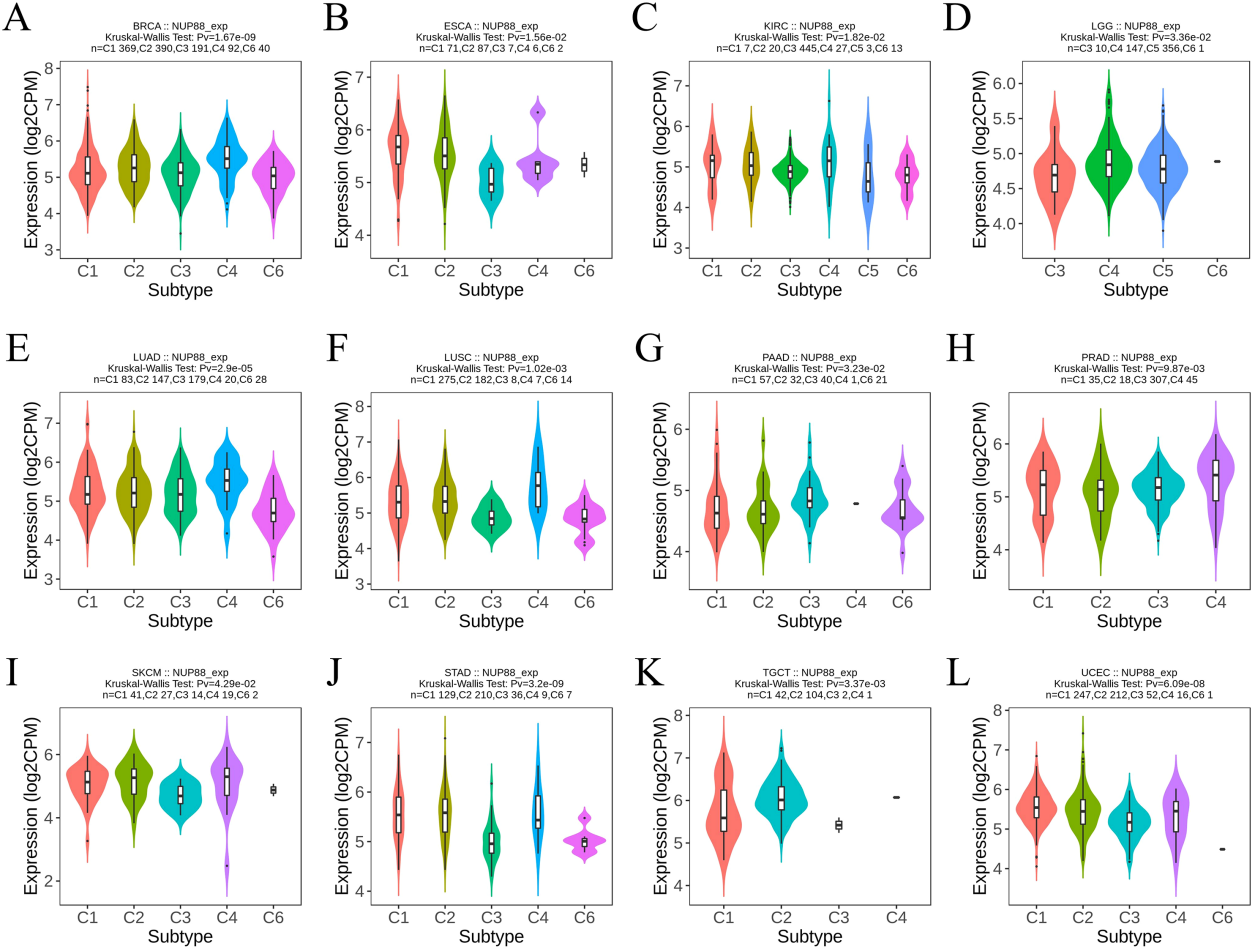
The relationships between NUP188 and immune subtypes in human cancers. **(A)** BRCA. **(B)** ESCA. **(C)** KIRC. **(D)** LGG. **(E)** LUAD. **(F)** LUSC. **(G)** PAAD. **(H)** NUP188. **(I)** SKCM. **(J)** STAD. **(K)** TGCT. **(L)** UCEC.

**Figure 7:**
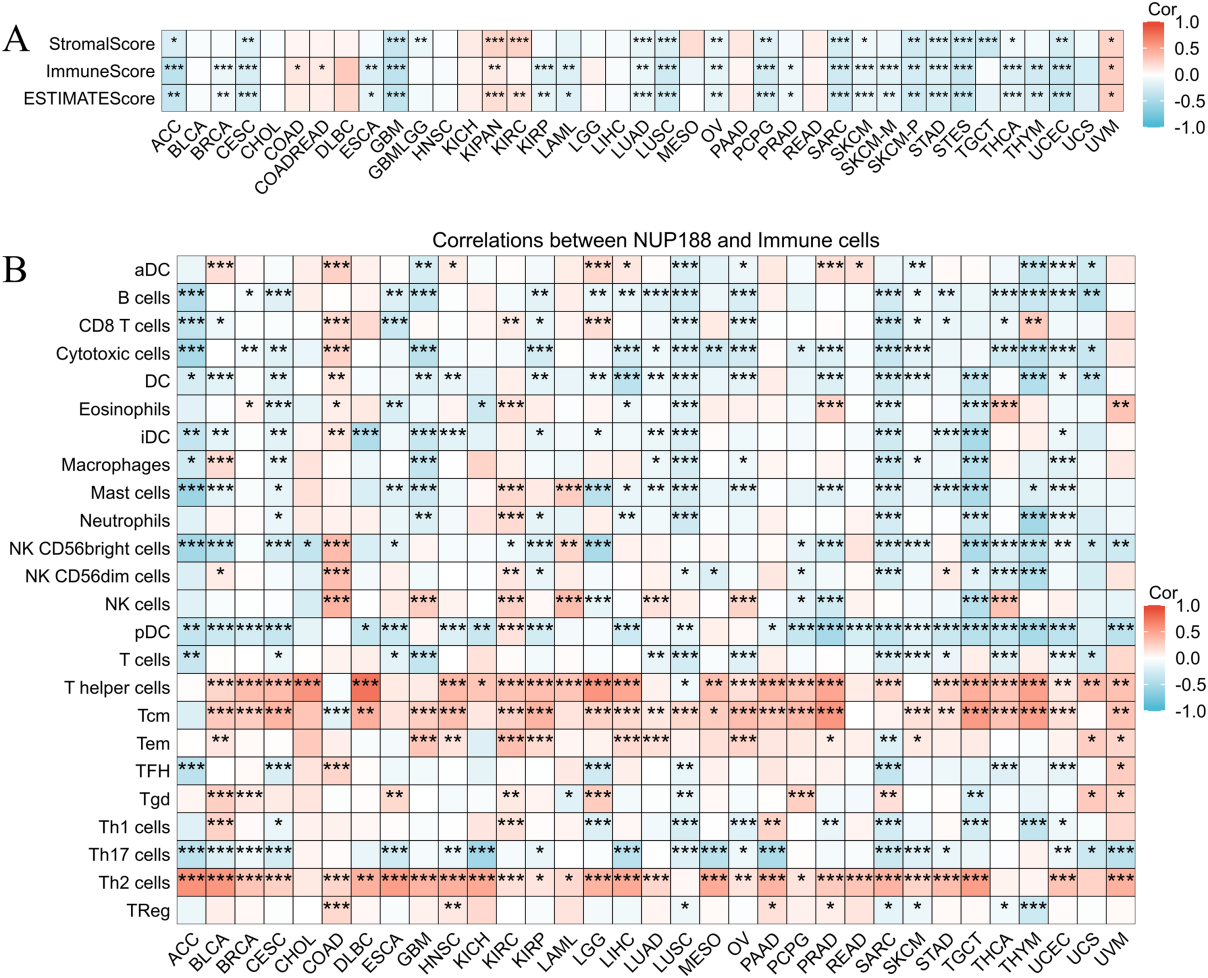
The role of NUP188 in pan-cancer immunoregulation. **(A)** The ESTIMATE analysis of NUP188 in pan-cancer. B The crorelations between NUP188 and immune cells. *P<0.05, **P<0.01, ***P<0.001.

The immune environment is known to play a vital role in regulating cancer progression and patients’ prognosis. The TISIDB database confirmed that different immune subtypes showed different NUP188 levels in 12 cancer types (**Figure 6**). To further prove the role of NUP188 in regulating the tumor microenvironment, the connection between NUP188 and three scores of ESTIMATE analysis was demonstrated. The result showed that NUP188 was negatively associated with immune score in 22 cancers and only positively in 4 cancers (**Figure 7A**). The results above indicated that NUP188 might inhibit the infiltrating immune cells in most cancer types.

Subsequently, we explored the correlations between NUP188 and 24 kinds of immune cells. As shown in **Figure 7B**, NUP188 was negatively associated with B cells in 18 cancers, with cytotoxic cells in 18 cancers, DC in 18 cancers, pDC 23 cancers, Th17 cells in 17 cancers. In addition, NUP188 showed positive connections to T helper cells in 25 cancers, to Tcm in 24 cancers, and Th2 cells in 26 cancers (**Figure 7B**).

### 3.6 The expression and prognostic value of NUP188 in GC patients

**Figure 8:**
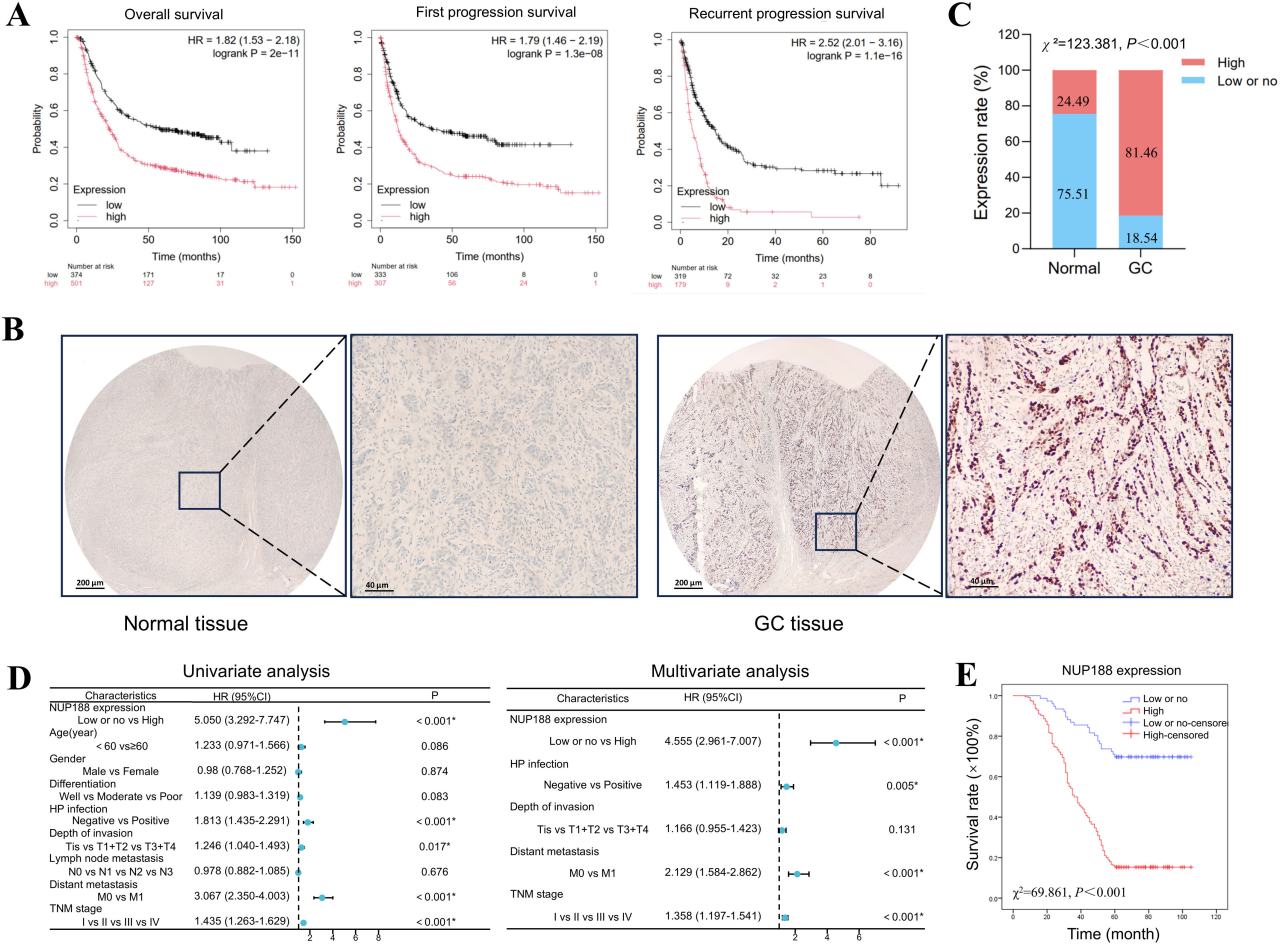
The association between NUP188 expression and GC prognosis. **(A)** The survival curves of GC patients with different NUP188 expression in Kaplan-Meier Plotter platform. **(B)** IHC staining for NUP188 expression in gastric samples. Left, normal gastric tissue with low NUP188 expression. Right, GC tissue with high NUP188 expression. **(C)** Statistical diagram for NUP188 protein in gastric samples. **(D)** Univariate and multivariate analysis of the prognostic factors for GC patients. **(E)** The relationship between NUP188 and overall survival of GC patients. *P<0.05.

The Kaplan-Meier Plotter demonstrated that GC patients with a higher NUP188 level tended to have shorter overall survival, first progression survival, and recurrent progression survival (**Figure 8A**). IHC was performed to confirm further the NUP188 expression in GC, including 98 paracancerous normal tissues and 410 GC tissues. As shown in **Figure 8B**, NUP188 was mainly expressed in the nucleus. High NUP188 expression was more common in GC tissues (334/410, 81.46%) than that in para-carcinoma tissues (24/98,24.49%) (χ^2^ = 123.381, *P*<0.001, **Figure 8C**).

As shown in **Table 1**, high NUP188 expression was significantly associated with positive H pylori infection (χ^2^ = 3.925, *P*=0.048), more profound depth of invasion (χ^2^ = 9.189, *P*=0.002), distant metastasis (χ^2^ = 8.544, *P*=0.003), and poor TNM stage (χ^2^ = 31.613, *P*<0.001). However, there was no connection between NUP188 and gender, age, differentiation, or lymph node metastasis (*P*>0.05). In univariate analysis, overall survival was significantly correlated to NUP188 (HR=5.050; 95% CI, 3.292–7.747; *P*<0.001) (**Figure 8D**). In multivariate analysis, NUP188 (HR=4.555; 95% CI, 2.961–7.007; *P*<0.001) was an independent prognostic factor for GC patients (**Figure 8D**). The clinical data of these 410 GC patients also confirmed that high NUP188 expression might predict poor overall survival (Figure E).

**Table 1:** The association between NUP188 expression and clinicopathological characteristics of GC patients.

Characteristics	n	NUP188 expression (%)		*χ* ²	*P*
		Low or no	High		
Total	410	76 (18.54)	334 (81.46)		
Gender				0.886	0.347
Female	122	26 (21.31)	96 (78.69)		
Male	288	50 (17.36)	238 (82.64)		
Age				0.001	0.990
<60	140	26 (18.57)	114 (81.43)		
≥60	270	50 (18.52)	220 (81.48)		
HP infection				3.925	0.048*
Positive	241	37 (15.35)	204 (84.65)		
Negative	169	39 (23.08)	130 (76.92)		
Differentiation				0.788	0.674
Well	117	23 (19.66)	94 (80.34)		
Moderate	164	27 (16.46)	137 (83.54)		
Poor	129	26 (20.16)	103 (79.84)		
Depth of invasion				9.189	0.002*
Tis+ T1+T2	66	21 (31.82)	45 (68.18)		
T3+ T4	344	55 (15.99)	289 (84.01)		
Lymph node metastasis				0.071	0.790
N0	80	14 (17.50)	66 (82.50)		
N1+N2+N3	330	62 (18.79)	268 (81.21)		
Distant metastasis				8.544	0.003*
M0	328	70 (21.34)	258 (78.66)		
M1	82	6 (7.32)	76 (92.68)		
TNM stage				31.613	<0.001*
I	26	15 (57.69)	11 (42.31)		
II	133	23 (17.29)	110 (82.71)		
III	129	25 (19.38)	104 (80.62)		
IV	122	13 (10.66)	109 (89.34)		

*P<0.05.

### 3.7 The effect of NUP188 knockdown on the biological behavior of GC cells

**Figure 9:**
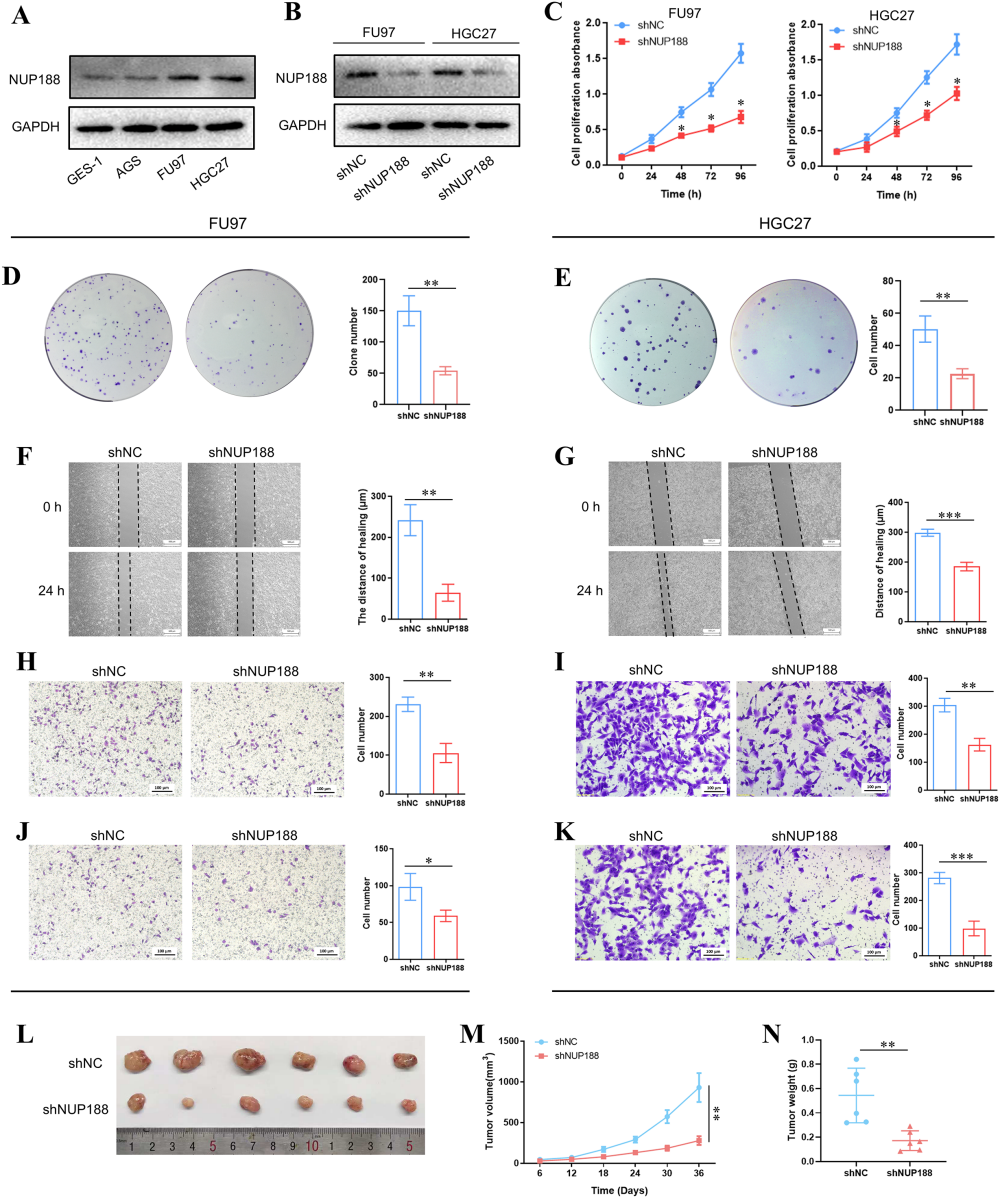
The influence of NUP188 knockdown on GC cells **(A)** The NUP188 expression in gastric cell lines. **(B)** The NUP188 level was detected by Western Blot after NUP188 knockdown. The cell proliferation was detected by CCK-8 assay **(C)** and clone formation assay **(D, E)**. The cell migration of FU97 **(F)** and HGC27 **(G)** was detected by wound healing assay. The cell migration of FU97 **(H)** and HGC27 **(I)** was detected by the Transwell migration assay. The cell invasion of FU97 **(J)** and HGC27 **(K)** was detected by the Transwell invasion assay. L The volumes of tumors in the xenograft mouse model. M The growth curves of tumors in the xenograft mouse model. N The weight of tumors in the xenograft mouse model. *P<0.05, **P<0.01, ***P<0.001.

Western Blotting demonstrated that the NUP188 level in GC cell lines was higher than in normal gastric epithelial cell line GSE-1, especially FU97 and HGC27 (**Figure 9A**). To explore the influence of NUP188 on GC cell behaviors, the NUP188 expression was interfered with in FU9 and HGC27 (**Figure 9B**). CCK-8 assay and clone formation assay showed that NUP188 interference inhibited the proliferation of GC cells (**Figures 9C–E**). Wound healing assay and Transwell migration assay demonstrated that downregulation of NUP188 could impair the migration capacity of GC cells (**Figures 9F–I**). Transwell invasion assay verified the decreased invasion capacity after NUP188 knockdown in GC cells (**Figures 9J–K**). *In vivo* experiment demonstrated that NUP188 knockdown inhibited the tumor growth in subcutaneous xenograft tumor model (**Figures 9L–N**). These results suggested that NUP188 knockdown could inhibit the GC cell proliferation, migration and invasion.

## 4 Discussion

The abnormal expressions of nucleoporins have been confirmed in multiple cancers. NUP93 is confirmed to be overexpressed in hepatocellular carcinoma (9), esophageal cancer (10), cervical cancer (11), and bladder cancer (12). NUP210 shows a higher expression in cervical cancer (13), prostate cancer (14), liver cancer (15), and meningiomas (16) than normal tissues. POM121 is upregulated in prostate cancer (17), colorectal cancer (18), gastric cancer (19), and lung cancer (20). However, the expression level of NUP188 in human cancer is little known. In this study, we compared the NUP188 mRNA and protein in different public databases, and confirmed that NUP188 was upregulated in most cancer types, but only downregulated in KICH. In addition, NUP199 presented good accuracy in distinguishing the tumor and normal tissues in 15 cancers according to the ROC method. Therefore, NUP188 might become a novel diagnostic biomarker for human cancers.

Different molecular subtypes often affect clinical characteristics and therapy outcomes of cancer patients (21). We explored the NUP188 expression in various kinds of molecular subtypes of human cancers. We found that NUP188 showed significant differences in different molecular subtypes of 10 cancer types. Furthermore, patients with different pathologic stages of ACC, KICH, KIRP, LIHC, and TGCT had different NUP188 levels. These results indicated that NUP188 might affect the tumor progression and patient prognosis. Subsequently, NUP188 was confirmed to be a prognostic factor for OS in 9 cancer types. To remove the interference of the nontumor factor, the relation between NUP188 and DSS and DFI was calculated, and the result demonstrated that NUP188 significantly affects the DSS and DFI in 6 cancers and 9 cancers, respectively. The results above indicated that NUP188 had the potential to predict the prognosis of cancer patients.

The gene functions of NUP188 were then explored through multiple databases. The GO/KEGG analysis of NUP188-related proteins confirmed that NUP188 was associated with nuclear pore assemble and nucleocytoplasmic transport, which was consistent with the common sense. The data from the GSCALite database demonstrated that NUP188 activated the apoptosis, cell cycle and DNA damage in a significant percentage of cancer types, indicating that NUP188 might play a different role in different cancers. NUP188 also inhibited the hormone AR/ER signaling pathways in some cases, meaning that NUP188 might work as a cancer suppressor in breast and prostate cancer. Receptor tyrosine kinases (RTKs) include epidermal growth factor receptors (EGFRs), vascular endothelial growth factor receptors (VEGFRs), platelet-derived growth factor receptors (PDGFRs), insulin-like growth factor receptors (IGFRs) which are related to tumor progression (22). The inhibition influence of NUP188 on the RTK pathway might elucidate the mechanism of anticancer action in some cancer types. On the single-cell level of each cancer, NUP188 also has a dual role in biological behavior regulation. For example, NUP188 was positively associated with inflammation, metastasis, and differentiation in LUAD, but showed negative relationship with these processes in BRCA.

Tumorigenesis is always derived from genomic variation, and molecular testing for genome is becoming an inseparable part of cancer research. Through the cBioPortal database, we found that mutation was the most common genomic variation associated with NUP188, and there were 255 mutation sites in the 1749 amino acids of NUP188. Take gastric cancer as an example, we confirmed that TTN and TP53 were the most frequent mutations related to NUP188, and these two gene mutations are closely linked to the progression and prognosis of GC (23, 24). Neo refers to a type of antigen that is produced due to genetic mutations in tumor cells, and the immune system recognizes tumor cells primarily by recognizing Neo on the surface of tumor cells, which are produced by genetic mutations in tumor cells. TMB refers to the number of somatic mutations contained in every million base pairs of the tumor cell genome. High TMB means that tumor cells have more genetic mutations, which is likely to produce more Neo, and tumor patients may be more likely to benefit from immunotherapy (25). MSI is mainly caused by dysfunction of the DNA mismatch repair system, manifesting as abnormal changes in the length of microsatellite sequences in the genome. Cancer cells with high MSI can also generate a large number of Neo, and a combination of TMB and MSI can more accurately predict the response rate of tumor immunotherapy (26). This study explored the relationship between NUP188 and TMB, Neo and MSI, and demonstrated that NUP188 showed positive connections to all these three indexes in COAD and UCEC, which meant that high NUP188 expression might be an immunotherapeutic indication for COAD or UCEC patients. MATH is an indicator used to quantify tumor heterogeneity, mainly assessing the diversity of tumor cell clones by analyzing the distribution characteristics of the frequency of mutant alleles in tumor samples (27). LOH refers to a state in which one of the two alleles at a specific genetic locus in a cell undergoes deletion or functional loss, resulting in the locus retaining only one functional allele (28). HRD refers to a state in which cells, due to functional abnormalities in the homologous recombination repair pathway, are unable to accurately repair DNA double-strand breaks, thereby inducing genomic instability (29). Furthermore, MATH, HRD and LOH are important indexes for genomic heterogeneity, and are associated with tumor prognosis and immunotherapy (27–29). This study demonstrated that NUP188 was also significantly connected to MATH, HRD and LOH in multiple cancers. These results suggested that NUP188 might regulate the tumor microenvironment (TME) by influencing genomic stability, thereby modulating the immunotherapy response.

TME is a complex and dynamic ecosystem about tumor growth and survival (30–32). This research found that different immune subtypes of 12 cancers presented different NUP188 expressions, indicating that NUP188 may participate in TME regulation. The ESTIMATE algorithm is a novel method to evaluate tumor purity through the analysis of stromal and immune cells in TME (33). Low scores of these criteria always mean poor survival of cancer patients (34). Our results demonstrated that NUP188 was negatively associated with three ESTIMATE scores in most cancer types. We then calculated the correlations between NUP188 expression and the infiltrating level of immune cells. B cells usually play a positive immunomodulatory role by producing antibodies, participating in antigen presentation, and promoting the activation and proliferation of T cells (35). Dendritic cells (DCs) play an antitumor role through the tumor-associated antigen presentation and the activation of T cell immune response (36). CD56bright NK cells is a subgroup of NK cells with a strong cytokine production capacity, and present potent antitumor responses (37). Our results demonstrated that NUP188 was negatively associated with infiltrations of B cells, cytotoxic cells, DCs, immature DC (iDC), plasmacytoid DC (pDC), and CD56bright NK cells in most cancer types. Th2 cells and Th17 cells are different subgroups of T helper cells, and all play dual roles in cancer progression (38, 39). NUP188 showed positive association with T helper cells and Th2 cells, and presented negative correlation to Th17 in most cancer types. Therefore, the role of T helper cells in NUP188-mediated immune TME deserved experimental validation. In addition, NUP188 also exhibited positive connection to central memory T cells (Tcm) which could recognize and kill tumor cells (40). However, the antitumor influence of upregulated Tcm might be weakened in tumors with high NUP188 expression due to the lack of assistance of DCs and NK cells.

As a malignant tumor in the digestive system, GC ranks fourth in cancer mortality, with more than one million new cases yearly (41). The 5-year survival rate of GC patients with early stage is more than 60%, while that falls to less than 5% in patients with distant metastases (42). However, most GC patients have reached the middle and late stage at first diagnosis due to early nonspecific symptoms, and many patients have lost the opportunity for surgery. Therefore, it’s necessary to investigate novel biomarkers for early diagnosis and prognosis of GC patients. Existing literature has suggested that NUP188 may play an important role in gastric cancer (43). The bioinformatics analysis confirmed that NUP188 was upregulated in GC tissues and had a good accuracy for GC diagnosis. Furthermore, NUP188 expression was also closely associated with the prognosis of GC patients. This study detected the NUP188 protein in 98 pericarcinomatous normal tissues and 410 GC tissues. The results of IHC confirmed the overexpression of NUP188 in GC tissues. Furthermore, NUP188 expression was significantly associated with H. pylori infection, depth of invasion, distant metastasis, and TNM stage, indicating that NUP188 might promote the proliferation and invasiveness of GC cells. The GC patients with high NUP188 levels were also confirmed to have poorer overall survival than other patients. These results suggested that NUP188 had the potential to serve as a novel biomarker for GC diagnosis and prognosis.

With the deepening of the research on the molecular mechanism of GC pathogenesis, molecular targeted therapy, as a new and targeted therapeutic means, occupies an important position in the field of gastric cancer treatment. Molecular targets approved for clinical GC treatment include HER2, VEGF and VEGFR (44, 45). CLDN18.2 and FGFR2b are also promising and burgeoning candidates for GC therapy (46–48). However, molecular targeted therapy alone has limited efficacy in GC treatment, and it’s necessary to find more biological targets. We inhibited the NUP188 expression in GC cells, and found that NUP188 knockdown in GC cells significantly impaired the tumor lethality, including cell proliferation, migration and invasion. These results indicated that NUP188 might be a potential biomarker for GC targeted therapy.

In conclusion, NUP188 was overexpressed in multiple cancers, and may play a vital role in tumor diagnosis and prognosis. NUP188 might participate in regulating biological behaviors and genomic heterogeneity, and was connected to infiltrating immune cells and immunomodulation. This study about the role of NUP188 in pan-cancer provided the reference basis for the clinical application of NUP188-based therapy.

## Data Availability

The original contributions presented in the study are included in the article/**Supplementary Material.** Further inquiries can be directed to the corresponding author.

## References

[1] Kumar A Sharma P Gomar-Alba M Shcheprova Z Daulny A Sanmartín T. Daughter-cell-specific modulation of nuclear pore complexes controls cell cycle entry during asymmetric division. Nat Cell Biol. (2018) 20:432–42. doi: 10.1038/s41556-018-0056-9, PMID: 29531309 PMC6029668

[2] Lim KS Wong RW. Targeting nucleoporin POM121-Importin β Axis in prostate cancer. Cell Chem Biol. (2018) 25:1056–8. doi: 10.1016/j.chembiol.2018.09.003, PMID: 30241601

[3] Chow KH Factor RE Ullman KS. The nuclear envelope environment and its cancer connections. Nat Rev Cancer. (2012) 12:196–209. doi: 10.1038/nrc3219, PMID: 22337151 PMC4338998

[4] Gandhi T Nathalie E Heinz S Wolfram A. The nucleoporin Nup188 controls passage of membrane proteins across the nuclear pore complex. Cell Biol Int. (2010) 189:1129–42. doi: 10.1083/jcb.200912045, PMID: 20566687 PMC2894445

[5] Fujita A Tsukaguchi H Koshimizu E Nakazato H Miyake N. Homozygous splicing mutation in NUP133 causes Galloway–Mowat syndrome. Ann Neurol. (2018) 84:814–28. doi: 10.1002/ana.25370, PMID: 30427554

[6] Sandestig A Engstrm K Pepler A Danielsson I Stefanova M. NUP188 biallelic loss of function may underlie a new syndrome: nucleoporin 188 insufficiency syndrome? Mol Syndromology. (2019) 10:313–9. doi: 10.1159/000504818, PMID: 32021605 PMC6995945

[7] Bindea G Mlecnik B Tosolini M Kirilovsky A Waldner M Obenauf AC. Spatiotemporal dynamics of intratumoral immune cells reveal the immune landscape in human cancer. Immunity. (2013) 39:782–95. doi: 10.1016/j.immuni.2013.10.003, PMID: 24138885

[8] Kuai X Lv J Zhang J Xu M Ji J. Serpin family A member 1 is prognostic and involved in immunological regulation in human cancers. Int J Mol Sci. (2023) 24:11566. doi: 10.3390/ijms241411566, PMID: 37511325 PMC10380780

[9] Lin C-S Liang Y Su S-G Zheng Y-L Yang X Jiang N. Nucleoporin 93 mediates β-catenin nuclear import to promote hepatocellular carcinoma progression and metastasis - ScienceDirect. Cancer Lett. (2022) 526:236–47. doi: 10.1016/j.canlet.2021.11.001, PMID: 34767927

[10] Zhang J Xin Y Ling X Liang H Zhang L Fang C. Nucleoporin 93, a new substrate of the E3 ubiquitin protein ligase HECTD1, promotes esophageal squamous cell carcinoma progression. Hum Cell. (2024) 37:245–57. doi: 10.1007/s13577-023-01005-2, PMID: 37993750

[11] Xiaolan O Xiaoming H Shuaibin L Jianguo H Lina H. Expression of Nup93 is associated with the proliferation, migration and invasion capacity of cervical cancer cells. Acta Biochim Biophys Sin (Shanghai). (2019) 51:1276–85. doi: 10.1093/abbs/gmz131, PMID: 31774908

[12] Wang Z Zhang J Luo L Zhang C Huang X Liu S. Nucleoporin 93 regulates cancer cell growth and stemness in bladder cancer via wnt/β-catenin signaling. Mol Biotechnol. (2024) 67(5):2072–84. doi: 10.1007/s12033-024-01184-9, PMID: 38744786

[13] Rajkumar T Sabitha K Vijayalakshmi N Shirley S Selvaluxmy G. Identification and validation of genes involved in cervical tumourigenesis. BMC Cancer. (2011) 11:80. doi: 10.1186/1471-2407-11-80, PMID: 21338529 PMC3050856

[14] Sugiura M Sato H Okabe A Fukuyo M Mano Y Shinohara KI. Identification of AR-V7 downstream genes commonly targeted by AR/AR-V7 and specifically targeted by AR-V7 in castration resistant prostate cancer - ScienceDirect. Transl Oncol. (2021) 14:100915. doi: 10.1016/j.tranon.2020.100915, PMID: 33096335 PMC7581977

[15] Hong SH Son KH Ha SY Wee TI Choi SK Won JE. Nucleoporin 210 serves a key scaffold for SMARCB1 in liver cancer. Cancer Res. (2021) 81:356–70. doi: 10.1158/0008-5472.CAN-20-0568, PMID: 33239431

[16] Mukherjee S Biswas D Epari S Shetty P Moiyadi A Ball GR. Comprehensive proteomic analysis reveals distinct functional modules associated with skull base and supratentorial meningiomas and perturbations in collagen pathway components. J Proteomics. (2021) 246:104303. doi: 10.1016/j.jprot.2021.104303, PMID: 34174477

[17] Becker F Offermann A Roesch MC Joerg V Roth D Lubczyk V. Up-regulation of POM121 is linked to prostate cancer aggressiveness and serves as a prognostic biomarker. Urol Oncol. (2022) 40:380.e11–.e18. doi: 10.1016/j.urolonc.2022.05.019, PMID: 35725938

[18] Wang T Sun H Bao Y En R Tian Y Zhao W. POM121 overexpression is related to a poor prognosis in colorectal cancer. Expert Rev Mol Diagn. (2020) 20:345–53. doi: 10.1080/14737159.2020.1707670, PMID: 31858845

[19] Kang C Jia L Hao L Zhang N Liu Y Zhang L. POM121 promotes the proliferation and metastasis of gastric cancer via PI3K/AKT/MYC pathway. Am J Cancer Res. (2021) 13:485–97., PMID: 36895982 PMC9989611

[20] Guan L Zhang L Wang T Jia L Zhao K. POM121 promotes proliferation and metastasis in non-small-cell lung cancer through TGF-β/SMAD and PI3K/AKT pathways. Cancer biomark. (2021) 32:293–302. doi: 10.3233/CBM-210001, PMID: 34151840 PMC12500068

[21] Collisson EA Bailey P Chang DK Biankin AV. Molecular subtypes of pancreatic cancer. Nat Rev Gastroenterol Hepatol. (2019) 16:207–20. doi: 10.1038/s41575-019-0109-y, PMID: 30718832

[22] Butti R Das S Gunasekaran VP Yadav AS Kumar D Kundu GC. Receptor tyrosine kinases (RTKs) in breast cancer: signaling, therapeutic implications and challenges. Mol Cancer. (2018) 17:34. doi: 10.1186/s12943-018-0797-x, PMID: 29455658 PMC5817867

[23] Yang Y Zhang J Chen Y Xu R Zhao Q MUC4 GW. MUC16, and TTN genes mutation correlated with prognosis, and predicted tumor mutation burden and immunotherapy efficacy in gastric cancer and pan-cancer. Clin Transl Med. (2020) 10:e155. doi: 10.1002/ctm2.155, PMID: 32898332 PMC7443139

[24] Graziano F Fischer NW Bagaloni I Bartolomeo MD Ruzzo A. TP53 mutation analysis in gastric cancer and clinical outcomes of patients with metastatic disease treated with ramucirumab/paclitaxel or standard chemotherapy. Cancers. (2020) 12:2049. doi: 10.3390/cancers12082049, PMID: 32722340 PMC7465166

[25] Chan TA Yarchoan M Jaffee E Swanton C Quezada SA Stenzinger A. Development of tumor mutation burden as an immunotherapy biomarker: utility for the oncology clinic. Ann Oncol. (2019) 30:44–56. doi: 10.1093/annonc/mdy495, PMID: 30395155 PMC6336005

[26] Palmeri M Mehnert J Silk AW Jabbour SK Ganesan S Popli P. Real-world application of tumor mutational burden-high (TMB-high) and microsatellite instability (MSI) confirms their utility as immunotherapy biomarkers. ESMO Open. (2022) 7:100336. doi: 10.1016/j.esmoop.2021.100336, PMID: 34953399 PMC8717431

[27] Wu X Song P Guo L Ying J Li W. Mutant-Allele tumor heterogeneity, a favorable biomarker to assess intra-Tumor heterogeneity, in advanced lung adenocarcinoma. Front Oncol. (2022) 12:888951. doi: 10.3389/fonc.2022.888951, PMID: 35847947 PMC9286753

[28] Chun SK Fortin BM Fellows RC Habowski AN Verlande A Song WA. Disruption of the circadian clock drives Apc loss of heterozygosity to accelerate colorectal cancer. Sci Adv. (2022) 8:eabo2389. doi: 10.1126/sciadv.abo2389, PMID: 35947664 PMC9365282

[29] Zhou Z Ding Z Yuan J Shen S Jian H Tan Q. Homologous recombination deficiency (HRD) can predict the therapeutic outcomes of immuno-neoadjuvant therapy in NSCLC patients. J Hematol Oncol. (2022) 15:62. doi: 10.1186/s13045-022-01283-7, PMID: 35585646 PMC9118717

[30] Li X Gao Y Xu Z Zhang Z Zheng Y Qi F. Identification of prognostic genes in adrenocortical carcinoma microenvironment based on bioinformatic methods. Cancer Med. (2020) 9:1161–72. doi: 10.1002/cam4.2774, PMID: 31856409 PMC6997077

[31] Rana M Kansal R Chaib M Teng B Morrrison M Hayes DN. The pancreatic cancer immune tumor microenvironment is negatively remodeled by gemcitabine while TGF-β receptor plus dual checkpoint inhibition maintains antitumor immune cells. Mol Carcinogenesis. (2022) 61:549–57. doi: 10.1002/mc.23401, PMID: 35319799 PMC12289352

[32] Romain.Désert Giannone F Schuster C Baumert TF. Tumor microenvironment-derived serum markers as a new frontier of diagnostic and prognostic assessment in biliary tract cancers. Int J Cancer. (2023) 152:804–6. doi: 10.1002/ijc.34357, PMID: 36455586 PMC7615303

[33] Yoshihara K Shahmoradgoli M Martínez E Vegesna R Kim H Torres-Garcia W. Inferring tumour purity and stromal and immune cell admixture from expression data. Nat Commun. (2013) 4:2612. doi: 10.1038/ncomms3612, PMID: 24113773 PMC3826632

[34] Yue Y Zhang Q Sun Z. CX3CR1 acts as a protective biomarker in the tumor microenvironment of colorectal cancer. Front Immunol. (2021) 12:758040. doi: 10.3389/fimmu.2021.758040, PMID: 35140706 PMC8818863

[35] Ni Z Xing D Zhang T Ding N Xiang D Zhao Z. Tumor-infiltrating B cell is associated with the control of progression of gastric cancer. Immunol Res. (2021) 69:43–52. doi: 10.1007/s12026-020-09167-z, PMID: 33236222

[36] Prete AD Sozio F Barbazza I Salvi V Tiberio L Laffranchi M. Functional role of dendritic cell subsets in cancer progression and clinical implications. Int J Mol Sci. (2020) 21:3930. doi: 10.3390/ijms21113930, PMID: 32486257 PMC7312661

[37] Wagner JA Rosario M Romee R Berrien-Elliott MM Fehniger TA. CD56bright NK cells exhibit potent antitumor responses following IL-15 priming. J Clin Invest. (2017) 127:4042–58. doi: 10.1172/JCI90387, PMID: 28972539 PMC5663359

[38] Basu A Ramamoorthi G Albert G Gallen C Beyer A Snyder C. Differentiation and regulation of TH cells: A balancing act for cancer immunotherapy. Front Immunol. (2021) 12 669474. doi: 10.3389/fimmu.2021.669474, PMID: 34012451 PMC8126720

[39] Karpisheh V Ahmadi M Abbaszadeh-Goudarzi K Saray MM Barshidi A Mohammadi H. The role of Th17 cells in the pathogenesis and treatment of breast cancer. Cancer Cell Int. (2022) 22:108. doi: 10.1186/s12935-022-02528-8, PMID: 35248028 PMC8897940

[40] Casati A Varghaei-Nahvi A Feldman SA Assenmacher M Rosenberg SA Dudley ME. Clinical-scale selection and viral transduction of human nave and central memory CD8 + T cells for adoptive cell therapy of cancer patients. Cancer Immunol Immunother. (2013) 62:1563–73. doi: 10.1007/s00262-013-1459-x, PMID: 23903715 PMC6348480

[41] Sung H Ferlay J Siegel RL Laversanne M Soerjomataram I Jemal A. Global cancer statistics 2020: GLOBOCAN estimates of incidence and mortality worldwide for 36 cancers in 185 countries. CA Cancer J Clin. (2021) 71:209–49. doi: 10.3322/caac.21660, PMID: 33538338

[42] Yuan L Xu ZY Ruan SM Mo S Cheng XD. Long non-coding RNAs towards precision medicine in gastric cancer: Early diagnosis, treatment, and drug resistance. Mol Cancer Ther. (2020) 19:96. doi: 10.1186/s12943-020-01219-0, PMID: 32460771 PMC7251695

[43] Jili H Weiqiang Y Yuting D Can L Yongkang W Qingfa W. A deep neural network for gastric cancer prognosis prediction based on biological information pathways. J Oncol. (2022) 2022:2965166. doi: 10.1155/2022/2965166, PMID: 36117847 PMC9481367

[44] Shitara K Bang YJ Iwasa S Sugimoto N Ryu MH Sakai D. Trastuzumab deruxtecan in HER2-positive advanced gastric cancer: exploratory biomarker analysis of the randomized, phase 2 DESTINY-Gastric01 trial. Nat Med. (2024) 30:1933–42. doi: 10.1038/s41591-024-02992-x, PMID: 38745009 PMC11271396

[45] Yin B Luo W. Efficacy and safety of neoadjuvant bevacizumab plus chemotherapy in locally advanced gastric cancer patients: a retrospective, comparative study. World J Surg Oncol. (2025) 23:26. doi: 10.1186/s12957-024-03624-x, PMID: 39875999 PMC11773743

[46] De Moraes FCA Pasqualotto E Chavez MP Ferreira ROM De Castria TB Burbano RMRJBC. Efficacy and safety of Zolbetuximab plus chemotherapy for advanced CLDN18.2-positive gastric or gastro-oesophageal adenocarcinoma: a meta-analysis of randomized clinical trials. BMC Cancer (2024) 24:240. doi: 10.1186/s12885-024-11980-w, PMID: 38383390 PMC10882870

[47] Wainberg ZA Enzinger PC Kang YK Qin S Yamaguchi K Kim IH. Bemarituzumab in patients with FGFR2b-selected gastric or gastro-oesophageal junction adenocarcinoma (FIGHT): a randomised, double-blind, placebo-controlled, phase 2 study. Lancet Oncol. (2022) 23:1430–40. doi: 10.1016/S1470-2045(22)00603-9, PMID: 36244398

[48] Narita Y Ogata T Ishizuka Y Sakakida T Wakabayashi M Kodama H. Trifluridine/tipiracil with and without ramucirumab for advanced gastric cancer: a comparative observational study. Sci Rep. (2024) 14:12658. doi: 10.1038/s41598-024-61975-7, PMID: 38830895 PMC11148118

