# Supplementary material for: The value of nucleoporin 188 in diagnosis, prognosis and immunoregulation: from pan-cancer analysis to gastric cancer verification
Source: Front Immunol. 2025 Aug 8;16:1586784. doi: 10.3389/fimmu.2025.1586784 (PMC12370645; doi:10.3389/fimmu.2025.1586784)
Supplement: Supplementary file 1 [file DataSheet1.docx]

**Supplementary methods**

**1. NUP188 knockdown**

The human GC cell lines FU97 and HGC27 were purchased from KeyGEN Biotechnology. The control shRNA (shNC) and the shRNA against NUP188 (shNUP188) (GeneCopoeia, China) were transfected into two cells using Lipofectamine 2000 (#11668019, ThermoFisher, USA) according to the manufacture. Two days later, the cells were used for the next experiment.

**2.** **Western Blot**

The protein was extracted from cell lines using RIPA buffer (#89901, ThermoFisher, USA). The protein samples were separated by sodium dodecyl sulfate polyacrylamide gel electrophoresis, and transferred into PVDF membrane. After blocking with 5% skim milk, the membrane was incubated with NUP188 Polyclonal Antibody (1:1000, #PA5-48940, ThermoFisher, USA) or Anti-GAPDH antibody (1:1000, #ab181602, Abcam, USA) overnight at 4℃. Then, the membrane was incubated with Goat Anti-Rabbit IgG H&L (HRP) (1:1000, #ab6721, Abcam, USA). The protein bands were detected by a ChemiDoc XRS^+^ system (Bio-Rad, USA).

**3.** **CCK-8 assay**

The cells were cultured in 96-well plates at a density of 2×10^4^ cells/ well. The original medium was replaced by the mixture of CCK-8 regent (#CK04, Dojindo, Japan) and DMEM medium (1:9) every 24 h. The cells were incubated at 37℃ and 5%CO_2_ for 2 h, and the absorbance in OD450 was detected.

**4.** **Clone formation assay**

The cells of each parallel group were planted in the 6-well plates at the same density (600 cells/ well for FU97 and 400 cells/ well for HGC27). Then the cells were cultured at 37℃ and 5%CO_2_ for 1 week. Finally, the cells were soaked in methanol and 0.1% crystal violet solution for 30 min, respectively. The stained cell clones containing 50 or more cells were counted.

**5. Wound healing assay**

The cells were cultured in a 6-well plate without fetal bovine serum (FBS). When the cell density reached 90%, a horizontal line was drawn with a tip. The scratches were photographed under a microscope at 0 h and 24 h, respectively.

**6.** **Transwell assay**

The Transwell chamber was covered with diluted matrigel basement membrane matrix (#356234, BD Biosciences, USA) for invasion assay (This step was unnecessary for migration assay). The cells were cultured in Transwell chambers (3×10^4^/ well) with serum-free DMEM medium, and the chambers were placed in a 24-well plate with DMEM medium containing 20% FBS. After a certain time (24 h for migration assay and 48 h for invasion assay), cells in the chamber were wiped gently, and the chambers were soaked in methanol and 0.1% crystal violet solution for 30 min, respectively. The stained cells residual on the lower surface of the chambers were counted.

**7.** **Subcutaneous xenograft tumor model**

Twelve 6-week-old male BALB/c nude mice were purchased from Charles River Laboratories (Beijing, China), and divided into two groups with 6 mice per group. HGC27-shNC and HGC27-shNUP188 were prepared into cell suspension (5×10^7^ cells /ml). Then the cell suspension (100 μl/ mouse) was injected subcutaneously into the right armpit of nude mice, and the size of subcutaneous tumor was measured every 6 days. The nude mice were sacrificed after 36 days, and the subcutaneous tumor was bluntly separated with elbow forceps.

**8. Statistical analysis**

All data were analyzed using SPSS 19.0 software (SPSS, USA). The correlation between NUP188 and clinicopathological features was calculated by the Pearson χ^2^ test. Kaplan-Meier method was used to analyze the cumulative survival rate. The Cox proportional hazard model was established, and univariate and multivariate analysis was performed to determine prognostic factors. *P*<0.05 was considered statistically significant.
